# Update in the treatment of extracranial atherosclerotic disease for stroke prevention

**DOI:** 10.1136/svn-2019-000261

**Published:** 2019-11-07

**Authors:** Zhu Zhu, Wengui Yu

**Affiliations:** 1 Department of Neurology, University of California Irvine, Irvine, California, USA; 2 Department of Neurology, Huashan Hospital Fudan University, Shanghai, China

**Keywords:** atherosclerosis, intervention, stroke

## Abstract

Stroke is a leading cause of adult mortality and disability worldwide. Extracranial atherosclerotic disease (ECAD), primarily, carotid artery stenosis, accounts for approximately 18%–25% of ischaemic stroke. Recent advances in neuroimaging, medical therapy and interventional management have led to A significant reduction of stroke from carotid artery stenosis. The current treatment of ECAD includes optimal medical therapy, carotid endarterectomy (CEA) and carotid artery stenting (CAS). The selection of treatments depends on symptomatic status, severity of stenosis, individual factors, efficacy and risk of complications. The aim of this paper is to review current evidence and guidelines on the management of carotid artery stenosis, including the comparison of medical and interventional therapy (CAS and CEA), as well as future directions.

## Introduction

Stroke is the leading cause of adult mortality and disability worldwide. Extracranial atherosclerotic disease (ECAD), primarily, carotid artery stenosis, accounts for approximately 18%–25% of ischaemic stroke.^1 2^ ECAD can be managed with optimal medical therapy (OMT), carotid endarterectomy (CEA), and carotid artery stenting (CAS). Treatment options largely depend on the presence of symptoms, severity of stenosis, individual factors, efficacy and risk of complications.

## Symptoms and severity of carotid stenosis

Symptomatic carotid artery stenosis is defined as focal neurological symptoms that are sudden in onset and referable to ipsilateral carotid atherosclerotic pathology, including one or more transient ischaemic attack (TIA) or ischaemic stroke within the previous 6 months.^3^ The risk of recurrent ipsilateral stroke in patients with symptomatic moderate to severe carotid stenosis varies from 2.7% within the first day to 18.8% within 90 days after symptoms onset,^4^ significantly higher than those with asymptomatic stenosis with annual risk of stroke ranging from 0.34% to 2%.^5^ Despite conflicting results on the association between severity of carotid artery stenosis and risk of stroke,^4 6^ linear correlation between the benefit from CEA and degree of stenosis has been confirmed by previous research. Data of 6092 patients with 35 000 patient-years of follow-up showed that the absolute risk reduction (ARR) from CEA was −2.2% in patients with <30% stenosis, 3.2% with 30%–49% stenosis, 4.6% with 50%–69% stenosis and 16.0% with 70%–99% stenosis.^7^ Therefore, the presence of symptoms and severity of stenosis serve as main factors for selection of treatment.

## Individual factors

### Age

Subgroup analysis of Carotid Revascularisation Endarterectomy vs Stent Trial (CREST) showed increased periprocedural stroke/MI/death by 1.77 times in patients older than 70 years treated with CAS, whereas no evidence of increased risk in CEA-treated patients.^8^ A meta-analysis of 4 randomised controlled trials (RCTs) also demonstrated significantly increased risk of stroke or death within 30 days after CAS in patients older than 70 and 80 years of age compared with those under 60 years of age (OR, 4.01 and 4.15, respectively).^9^ This association, however, was not found in patients undergoing CEA. Notably, even though CEA may be generally preferable to CAS in patients over 70 years old due to lower periprocedural rate of stroke or death,^9^ CAS is a reasonable choice in elderly patients with unfavourable anatomy for CEA, radiation-induced stenosis or restenosis after CEA.

### Gender

Pooled data from ECST (European Carotid Surgery Trial) and NASCET (North American Symptomatic Carotid Endarterectomy Trial) found greater benefit from surgery in men with the number needed to treat to prevent ipsilateral stroke in 5 years being 9 for men vs 36 for women in patients with 50% or higher stenosis.^9^ In addition, the 30-day perioperative risk of death was significantly higher in women than in men (2.3% vs 0.8%, p=0.002).^10^ Combined analysis of NASCET and the ASA and Carotid Endarterectomy (ACE) trial found no benefit from CEA in women (ARR=3.0%, p=0.94), contrary to men (ARR=10.0%, p=0.02) in 50%–69% carotid stenosis. In contrast, with 70% to 99% stenosis, CEA was beneficially in both men and women with similar 5-year ARR in stroke (17.3% vs 15.1%).^10^ Therefore, CEA is effective for stroke prevention in symptomatic severe carotid stenosis (≥70%) regardless of genders, while may be only beneficial in men and selected women (eg, high risk of stroke) with moderate stenosis (50%–69%).

### Lesion features

Signs of unstable plaques—including rapidly progressing lesions, intraplaque haemorrhage, irregular/ulcerated surface, inflammation and microvascularization—have been increasingly reported as an independent predictor of stroke.^1 11–14^ Latest European Society of Cardiology (ESC) guideline also recommends targeting revascularisation in a subgroup of patients with risky clinical and/or imaging features, including ipsilateral silent infarction, stenosis progression, large plaques, echolucent plaques, lipid-rich necrotic core and so on.^15^

### Bilateral carotid stenosis

Various degrees of bilateral carotid stenosis are not rare in patients with atherosclerotic disease. For severe stenosis, staged rather than simultaneous approach is recommended due to risks of respiratory failure or fluctuating blood pressure.^16^ If surgery is indicated, then the symptomatic side is generally treated first. For bilateral asymptomatic stenosis, more severe stenosis is recommended to be addressed first. If the degree of stenosis is similar on both sides, then the artery supplying the dominant hemisphere can be considered for treatment first. Analysis of NASCET showed higher periprocedural complications of CEA in patients with contralateral carotid occlusion,^17^ while the outcome after CAS seemed to be less affected according to a review of 1375 patients.^18^

### Tandem lesions

The reported prevalence of stenosis of the internal carotid artery and ipsilateral common carotid is 4.3%.^19^ Treatment of tandem lesions is challenging with up to 20% perioperative mortality rate with CEA.^20^ Hybrid repair comprising CEA of the carotid bifurcation and retrograde endovascular repair of common carotid artery has been frequently reported with lower combined stroke and death rate than CEA alone.^21^ Research with a small sample size has also reported the use of endovascular therapy for the treatment of tandem lesions.^22^

### Chronic carotid artery occlusion

Patients with symptomatic chronic carotid artery occlusion and haemodynamic cerebral ischaemia are at high risk for subsequent stroke when treated medically.^23^ However, the Carotid Occlusion Surgery Study (COSS) showed that EC-IC bypass surgery plus medical therapy compared with medical therapy alone did not reduce the risk of recurrent ipsilateral ischaemic stroke at 2 years. Medical treatment continues to be the current standard of care for carotid occlusion. Recently, emerging small sample studies have demonstrated the efficacy of reopening of chronically occluded carotid artery.^24–26^ These studies indicate that the reopening of chronic carotid artery occlusion may be effective for patients with chronic carotid artery occlusion. However, randomised clinical trials are required to confirm the safety and benefit. In addition to treating culprit artery, contralateral CEA has been reported in patients with carotid occlusion and compromised cerebral haemodynamic reserve.^27^

Some other reported factors include type of symptoms (TIA, minor or major stroke; ocular or hemispheric symptoms), time since last symptomatic event and recurrence of symptoms.^28^

## Medical management

Patients with ECAD can benefit from OMT consisting of antiplatelet agents, stains, and risk factor control.^29^

(1) Antiplatelet agents: although the benefit of single antiplatelet agent for stroke prevention in asymptomatic carotid stenosis has not been confirmed by RCTs,^30^ current guidelines recommend lifelong low-dose aspirin as part of OMT to reduce the risk of stroke and other cardiovascular events.^15^ Dual antiplatelet therapy has been recommended during the periprocedural period and for at least 1 month after CAS.^31^

(2) Statins: statins have been routinely used in RCTs and clinical settings. A meta-analysis of 26 studies reported efficacy of statin with a dose-dependent protective effect,^32^ which was consistent with findings from 2 RCTs done afterwards.^33 34^

(3) Risk factor control: hypertension is an important risk factor for ECAD, and the goal of blood pressure (BP) in non-diabetic patients with asymptomatic carotid stenosis is recommended below 140/90 mm Hg.^35^ Patients with concomitant diabetes are at particularly increased risk of cerebrovascular events, for whom a diastolic BP ≤85 mm Hg has been recommended by the latest ESC guidelines.^15^

Previous studies have shown up to 26% risk of ipsilateral ischaemic stroke over 2 years in patients with symptomatic severe carotid artery stenosis despite OMT.^5^ It is therefore pivotal to consider more effective intervention.

## Interventional management

Interventional management consisting mainly of CEA and CAS has been shown to decrease the stroke rate in patients with carotid artery stenosis.^3 24 25 31–35^

### Carotid endarterectomy

ECST, NASCET and VA309 (Veterans Affairs 309) trials have demonstrated significant benefit of surgical intervention over medical treatment for secondary stroke prevention in patients with ipsilateral 50%–99% symptomatic carotid artery stenosis, with maximal efficacy in patients with 70%–99% carotid stenosis.^3 36 37^ Of note, pooled analysis of these trials showed no benefit of CEA for patients with 0%–49% stenosis.^7^

For asymptomatic carotid stenosis, ACAS (Asymptomatic Carotid Atherosclerosis Study) and ACST-1 (Asymptomatic Carotid Surgery Trial) established the benefit of CEA over medical therapy alone in patients with 60%–99% carotid stenosis.^38 39^ However, both studies started before the era of modern OMT, the widespread use of which has reduced the annual stroke rate significantly since the 1990s.^40^ In ACST-1, for example, the percentage of statin use has increased from 10% in the early period of recruitment to 80% by the end of follow-up.^41^ As such, it may be reasonable to consider OMT first for some patients who were considered surgical candidates in the past.

### CEA versus CAS

CEA was first described in 1975 by DeBakey and has since become a conventional treatment for severe ECAD.^42^ As an alternative to CEA, CAS emerged in 1989 and has proven to be effective and safe for carotid artery stenosis. A number of RCTs have been done to compare the two interventional therapies (table 1).^43–58^ Most studies have shown a higher rate of periprocedural stroke from CAS and a higher incidence of myocardial infarction (MI) with CEA. Similar findings have also been reported by a Cochrane review of 7572 patients, including 16 trials in 2012,^59^ and a meta-analysis of 6526 patients from 5 RCTs in 2017.^60^ Similar long-term outcomes, including the rate of ipsilateral ischaemic stroke or death with CAS and CEA, have been reported by most of the studies. CEA is preferable to CAS in patients over 70 years old.^9^

**Table 1:** RCTs to compare CEA and CAS for carotid stenosis

Trial	Study population	Follow-up	Primary endpoint	Results
WALLSTENT^56^	Symptomatic stenosis of 60%–99% (n=219)	24 hours; 1, 6, 12 months	Ipsilateral stroke, or death within 1 year	CAS with significantly higher primary endpoint (12.1% vs 3.6%, p=0.022) No significant difference in any major stroke at 1 year (3.7% vs 0.9%, p=0.204) CAS with significantly higher complication rates at 30 days (12.1% vs 4.5%, p=0.049)
CAVATAS^43 55^	Carotid stenosis equally suitable for CAS and CEA (n=504)	Median 5 years	Any stroke or death	No significant difference for disabling stroke or death within 30 days (6.4% vs 5.9%) CAS with significantly more severe restenosis after 1-y (14% vs 4%, p<0.001) CAS with higher 8-y rate of ipsilateral (11.3% vs 8.6%) & any stroke (21.1% vs 15.4%)
SAPPHIRE^46 47^	Symptomatic: >50%; Asymptomatic: >80%; (n=334)	30 days; 1, 2, 3 years	Death, stroke, or MI within 30 days; death or ipsilateral stroke beyond 30 days	Lower primary endpoint with CAS (12.2% vs 20.1%) (p=0.053) Less carotid revascularisation with CAS at 1 year (0.6% vs 4.3%, p=0.04) No significant difference in outcome at 3 years (24.6% vs 26.9%)
EVA-3S^50 51^	Symptomatic carotid stenosis of ≥60% (n=527)	Median 7.1 years	Composite of any stroke or death within 30 days	Significantly higher rate of any stroke or death with CAS within 30 days (9.6% vs 3.9%), at 6 months (11.7% vs 6.1%), and 5 years (11.0% vs 6.3%) No significant difference in any stroke or death at 10 years (11.5% vs 7.6%, p=0.07)
SPACE^48 49^	Symptomatic severe carotid stenosis (n=1200)	1, 7, 30 days; 6, 12, 24 months	ipsilateral ischaemic stroke or death within 30 days	Primary endpoint: CAS 6.84% vs CEA 6.34% (p*=*0.09 for non-inferiority) No significant difference in ipsilateral ischaemic stroke and periprocedural stroke or death at 2 years (CAS 9.5% vs CEA 8.8%) Significantly higher rate of restenosis with CAS (10.4% vs 4.6%, p=0.009)
CREST^44 45^	Symptomatic: >50% on angiography, >70% on CTA, MRA or US Asymptomatic: >60% on angiography, >70% on US, >80% on CTA or MRA (n=2502)	Median 2.5 years; 10 years	composite of stroke, MI or death during periprocedural period or ipsilateral stroke within 4 years after randomisation	No significant difference in primary endpoint: CAS 7.2% vs CEA 6.8% (p=0.51) Similar in periprocedural death: CAS 0.7% vs CEA 0.3% (p=0.18) Significantly more periprocedural stroke in CAS (4.1% vs 2.3%, p=0.01) Significantly more MI in CEA (2.3% vs 1.1%, p=0.03); No significant difference in primary endpoint at 10 years (CEA 9.9% vs CAS 11.8%) No significant difference in postprocedural stroke at 10 years (CEA 5.6% vs CAS 6.9%)
ICSS^52 53^	Symptomatic carotid stenosis of more than 50% (n=1713)	Median 4.2 years	3 year rate of fatal or disabling stroke in any territory	No significant difference in disabling stroke or death at 120 days (4.0% vs 3.2%) Higher incidence of stroke, death or procedural MI with CAS at 120 days (8.5% vs 5.2%, p=0.006) Higher risk of stroke (HR, 1.92) and all-cause death (HR, 2.76) with CAS at 120 days Similar 5 year risk of fatal or disabling stroke (6.4% vs 6.5%) Higher rate of any stroke at 5 years with CAS (15.2% vs 9.4%, p<0.001) No significant difference in mRS at 1 year, 5 years and final follow-up
ACT-1^54^	Asymptomatic severe carotid stenosis (n=1453)	5 years	Composite of death, stroke, or MI within 30 days or ipsilateral stroke within 1 year	No significant difference in primary endpoint (3.8% vs 3.4%) No significant difference in stroke or death within 30 days (2.9% vs 1.7%, p=0.33) No significant difference in ipsilateral stroke (2.2% vs 2.7%, p=0.51) and overall survival rate (87.1% vs 89.4%, p=0.21) from 30 days to 5 years Similar cumulative 5 year rate of stroke-free survival (93.1% vs 94.7%, p=0.44)

CAS, carotid artery stenting; CEA, carotid endarterectomy; CTA, CT angiography; MI, myocardial infarction;MRA, magnetic resonance angiography; mRS, modified Rankin Scale; RCT, randomi**s**ed controlled trial; US, ultrasonography.

## Current guidelines

The guideline recommendations for the management of symptomatic and asymptomatic carotid artery stenosis are listed in the online supplementary table. In general, current guidelines recommend OMT as an essential treatment for all patients with carotid artery stenosis, whereas symptomatic patients with >50% stenosis and highly selected asymptomatic patients with >60% stenosis be considered for additional interventional management if the estimated periprocedural complication rate is <3%.^50–52^ The choice between CEA and CAS should be made after considering demographics (eg, age and gender), anatomic, clinical (eg, contralateral TIA/stroke) and imaging (ipsilateral silent infarction, stenosis progression, spontaneous embolisation on transcranial Doppler, impaired cerebral vascular reserve, large plaques and so on) features.^2 50 51^

10.1136/svn-2019-000261.supp1 Supplementary data

## Future direction

Due to significant advances in medical therapy, risk reduction and endovascular technology in recent years, there is renewed discussion regarding the superiority of CEA over CAS and interventional management over the best medical therapy, especially in asymptomatic carotid stenosis. Several studies are being conducted to address these issues.

ACST-2 is an RCT comparing immediate and long-term safety and efficacy of CEA versus CAS in a patient with severe asymptomatic stenosis.^61^ The primary endpoint is 30-day MI, stroke and death, with subgroup analysis emphasising health economic aspects including procedural and stroke-related healthcare costs and quality of life. This study is recruiting patients from over 20 countries currently with 3600 patients planned to be enrolled by 2019.

SPACE 2 is a three-arm RCT designed to compare current OMT with CAS and CEA in addition to conservative treatments in patients with asymptomatic carotid artery stenosis. The study was halted after enrolling 513 patients. The 30-day rate of stroke/death was 2.54%, 1.97% and 0% in CAS, CEA and OMT groups, respectively.^62 63^

CREST-2 is an undergoing three-arm RCT to compare current OMT, OMT plus CEA, and OMT plus CAS for asymptomatic severe carotid stenosis, which enables a direct comparison of CAS and CEA. The primary endpoint is any stroke/death within 44 days after randomisation or ipsilateral ischaemic stroke within 4 years. This study is estimated to be completed by 2020.^64^

ECST-2 (ISRCTN 97744893) is an international RCT aimed to investigate the optimal treatment in patients with symptomatic or asymptomatic moderate or severe carotid stenosis at low or intermediate risk of stroke, in which patients will be randomised to OMT versus CAS or CEA. The primary endpoint is any stoke at any time or non-stroke death within 30 days after surgery. This trial is currently recruiting participants and estimated and estimated to be completed by 2022.
