# Supplementary material for: Update in the treatment of extracranial atherosclerotic disease for stroke prevention
Source: Stroke Vasc Neurol. 2019 Nov 7;5(1):65–70. doi: 10.1136/svn-2019-000261 (PMC7213501; doi:10.1136/svn-2019-000261)
Supplement: Supplementary data [file svn-2019-000261supp001.pdf]

Supplementary table. Current guidelines for treatment of symptomatic and asymptomatic carotid stenosis

Guidelines	Symptomatic status	OMT	CEA	CAS
ESC Guideline	symptomatic	Stenosis <50%	70–99% carotid stenosis (Class I, level A),<br>50–69% carotid stenosis (Class IIa, level A),<br>with documented procedural death/stroke rate < 6%;<br>Preferably performed within 14 days of symptom onset (Class I, level A)	High-risk anatomical features or medical comorbidities for CEA, with documented procedural death/stroke rate < 6% (Class IIa, level B);<br>Alternative to CEA in average surgical risk patients, with documented procedural death/ stroke rate < 6% (Class IIb, level B);<br>Preferably performed within 14 days of symptom onset (Class I, level A)
	asymptomatic	Stenosis < 60%;<br>Occlusion or near-occlusion;<br>Unfavorable anatomy or life expectancy ≤ 5 years	Average surgical risk patients with 60-99% stenosis and characteristics (clinical and/or imaging) associated with an increased risk of stroke, provided documented perioperative stroke/death rates are <3% and the patient's life expectancy is > 5 years (Class IIa, level B);	High risk for CEA patients with 60-99% stenosis and characteristics associated with an increased risk of stroke, provided documented perioperative stroke/death rates are <3% and the patient's life expectancy is > 5 years (Class IIa, level B);<br>Alternative to CEA in average surgical risk patients with 60-99% stenosis and characteristics associated with an increased risk of stroke, provided documented perioperative stroke/death rates are <3% and the patient's life expectancy is > 5 years (Class IIb, level B)
AHA/ASA guideline	symptomatic	Stenosis <50%: antiplatelet therapy, statin therapy, and risk factor modification is	70%–99% carotid stenosis, with perioperative morbidity and mortality risk estimated to be <6% (Class I, level A);	Alternative to CEA for average- or low-risk surgical patients with >70% stenosis by noninvasive imaging or >50% by catheter-based imaging or noninvasive imaging with corroboration and the anticipated rate

		recommended for all patients with carotid artery stenosis and a TIA or stroke (Class I, level A).	50%–69% carotid stenosis, in combination of patient-specific factors (e.g, age, sex, and comorbidities), with perioperative morbidity and mortality risk estimated to be <6% (Class I; Level B);<br>Performed within 2 weeks of neurological event if no contraindications to early revascularization (Class IIa, level B);<br>Preferred in patients > 70 years old; CAS is equivalent to CEA in younger patients (Class IIa, level B);<br>Performed by operators with established periprocedural stroke and mortality rates of <6% for symptomatic patients (Class I, level B);	of periprocedural stroke or death is <6% (Class IIa, level B);<br>CAS in equivalent to CEA in younger patients (Class IIa, level B);<br>>70% carotid stenosis with anatomic or medical conditions associated with increased risk for surgery or presence of other specific circumstances (such as radiation-induced stenosis or restenosis after CEA) (Class IIa, level B);<br>Performed by operators with established periprocedural stroke and mortality rates of <6% for symptomatic patients (Class I, level B);<br>Performed within 2 weeks of neurological event if no contraindications to early revascularization (Class IIa, level B);
	asymptomatic	Daily aspirin, statin, appropriate medical therapies and lifestyle changes for all patients (Class I, level C);<br>Aspirin perioperatively unless contraindicated (Class I, level C);<br>Effectiveness of CEA/CAS and OMT alone not well established (Class IIb, level B).	Considered in patients with >70% stenosis if risk of perioperative stroke, MI and death <3%. Effectiveness compared with OMT alone not established (Class A, level A);	Highly selected patients with carotid stenosis (minimum, 60% by angiography, 70% by validated Doppler ultrasound). Effectiveness compared with OMT alone not well established (Class IIb, level B);

ASA/ACCF/AHA/<br>AANN/AANS/<br>ACR/ASNR/CNS/<br>SAIP/SCAI/SIR/<br>SNIS/SVM/<br>SVS guideline	symptomatic	Patients at high risk of complications for CEA or CAS because of comorbidities;<br>Effectiveness of revascularization versus OMT alone not well established (Class IIb, level B);<br>Stenosis <50%;<br>Chronic total occlusion;<br>Severe disability from stroke.	>70% carotid stenosis and average or low surgical risk (Class I, level A) or<br>> 50% as documented by catheter angiography (Class I, level B) with estimated rate of perioperative stroke or mortality less than 6%;<br>When revascularization is indicated in older patients, particularly when arterial patho-anatomy is unfavorable for CAS (Class IIa, level B);<br>Performed within 2 weeks is preferable (Class IIa, level B).	Alternative to CEA in patients with >70% stenosis as documented by noninvasive imaging or >50% stenosis as documented by catheter angiography with average or low risk of complications associated with CAS, with anticipated rate of periprocedural stroke or mortality less than 6% (Class I, level B);<br>When revascularization is indicated in patients with neck anatomy unfavorable for arterial surgery (Class IIa, level B);<br>Performed within 2 weeks is preferable (Class IIa, level B).
	asymptomatic	Patients at high risk of complications for CEA or CAS because of comorbidities;<br>Effectiveness of revascularization versus OMT alone not well established (Class IIb, level B);<br>Stenosis <50%;<br>Chronic total occlusion;<br>Severe disability from stroke.	More than 70% stenosis, and risk of perioperative stroke, MI, and death is low (Class IIa, level A);	Considered in highly selected patients with stenosis (minimum 60% by angiography, 70% by validated Doppler ultrasound).<br>Effectiveness compared with OMT alone not well established (Class IIb, level B);
